# Beyond the WHO classification of meningioma: using molecular diagnostics to guide management

**DOI:** 10.47795/WVJZ9783

**Published:** 2023-08-08

**Authors:** Abigail Clynch, George E Richardson, Mohammad A Mustafa, Conor S Gillespie, Nitika Rathi, Ali Bakhsh, Rasheed Zakaria, Abdurrahman I Islim, Christopher P Millward, Michael D Jenkinson

## Abstract

Meningioma are the most common primary brain tumour. Classically, meningioma are phenotypically grouped using the World Health Organisation (WHO) classification system. However, it is now understood that the WHO approach overfits tumours into three grades, resulting in similarly graded tumours displaying phenotypically distinct behaviour. There is a growing body of research investigating the molecular biology of these tumours, including genomic, transcriptomic, metabolomic, proteomic, and methylomic profiling. Such advancements in molecular profiling of meningioma are providing greater accuracy in prognostication of tumours.

Furthermore, a clearer understanding of tumour molecular biology highlights potential targets for pharmacotherapies. Currently, the routine application of in-depth tumour molecular analysis is limited, however as it becomes more widely available it will likely result in improved patient care. This review seeks to explore the important developments in meningioma molecular biology, discussed in the context of their clinical importance.

## Introduction

Meningioma are the most common primary brain tumour, accounting for 38% of all Central Nervous System (CNS) neoplasms [1]. An association with increased age combined with a globally ageing population has resulted in an increased meningioma disease burden [2]. Other risk factors for meningioma development include ionising radiation, female sex and genetic disorders [2]. The management of symptomatic meningioma is surgical resection, with adjuvant fractionated radiotherapy and radiosurgery implemented in specific circumstances (e.g. sub-totally resected tumours) [2]. Following surgery, extent of tumour resection can be classified according to the Simpson grading system, or more broadly into gross- or sub-total resection. Key prognostic factors for recurrence include extent of resection, World Health Organisation (WHO) tumour grade, and use of adjuvant therapies [2].

**Figure 1:**
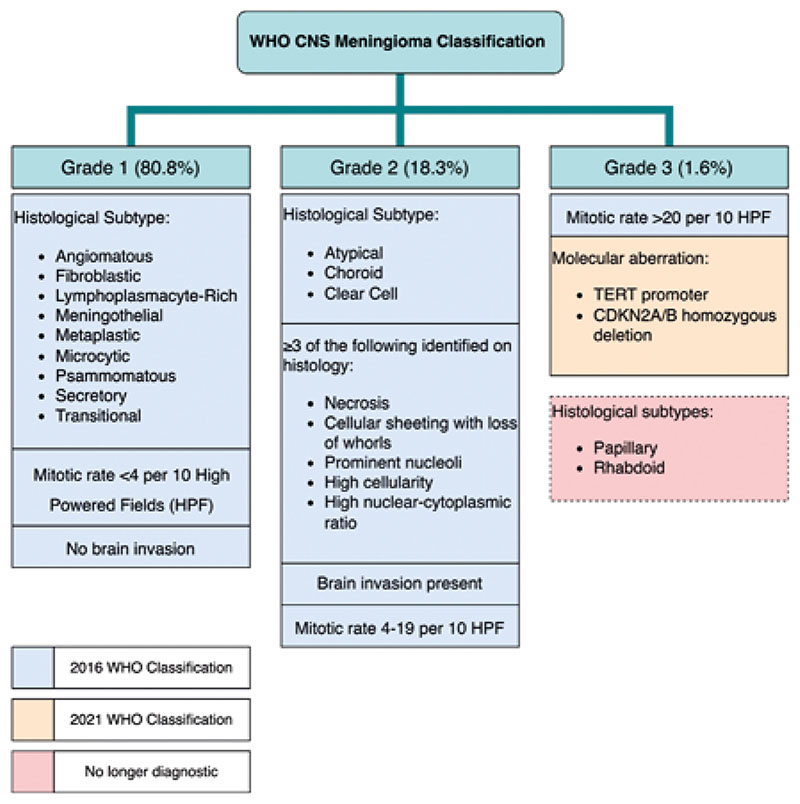
Diagram highlighting the CNS meningioma classification system. Box colour corresponds to year of inclusion in the classification and highlights criteria that are no longer considered diagnostic as of the 2021 update [3].

The WHO CNS tumour classification system (Figure 1) received its most recent update in 2021 [3]. The 2016 version primarily used histopathological findings to classify meningioma into grades 1, 2, and 3 [4, 5]. Grade 1 tumours are the most common and least aggressive, whilst grades 2 and 3 are rarer, more aggressive, tumours [1,2]. Survival rates vary between grades, with grade 1 being the highest (10 year relative survival 96.8%), then grade 2 (90.2%), and grade 3 having the lowest survival rates (30.4%) [6]. Notably, the 2021 update deviates from the previous system by the including a number of molecular factors. The presence of TERT promoter region mutations and CDKN2A/B homozygous deletion are now diagnostic of grade 3 meningioma [3,7,8]. Furthermore, the presence of rhabdoid and papillary histological sub-types are no longer independently diagnostic for WHO grade 3 meningioma [3].

The changes in the 2021 WHO classification reflect a paradigm shift in the field of neurooncology. Advancements in the genomic, transcriptomic, methylomic, proteomic, and metabolomic profiling have resulted in higher fidelity characterisation of CNS tumours. Despite the inclusion of prominent molecular factors, the current WHO classification does not fully represent the vast heterogeneity represented by the variable clinical behaviour seen in meningioma [2]. Advanced molecular characterisation of tumours also widens the potential for novel treatments by targeting the essential drivers of neoplastic growth. There are currently a number of targeted therapies for meningioma under investigation [9,10]. As our understanding of tumour biology grows, monitoring and treatment may be tailored to specific genetic aberrations, allowing intensification for aggressive phenotypes and relative sparing of benign acting tumours. Despite demonstrating superiority over the WHO classification, very few centres offer fully integrated molecular diagnostic approaches as routine clinical practice. This review highlights the prominent meningioma molecular factors being investigated and discusses their importance, as neuro-oncology moves ever closer towards a fully personalised medicine approach.

## Neurofibromatosis 2

Loss of chromosome 22 was the first recurrent genetic alteration found in meningioma [11]. Further examination of this alteration found loss of heterozygosity on chromosome 22q (LOH22). Coding for Merlin, NF2 is considered the most probable meningioma-associated gene located in this region and is found in 50-60% of meningioma [11–14]. Merlin is thought to link the actin cytoskeleton to the plasma membrane and acts as a tumour suppressor [15]. The exact biochemical mechanism by which Merlin works is not fully understood [16].

NF2 can be involved in hereditary neurofibromatosis type 2. Meningioma in this circumstance are described as NF2 associated Meningioma. Similarly, individuals without the condition can develop sporadic meningioma that contain a NF2 mutation – this is described as NF2 mutated meningioma. A substantial alteration in Merlin is needed to result in meningioma development, with mutational frequency increasing with WHO grade [13,14]. No NF2 hotspots have been located at present [13]. NF2 frequency varies across histopathological subtypes, for example it is significantly lower in meningothelial meningioma [14]. It has been proposed that separation into NF2 and non-NF2 meningioma could be a reasonable adjustment to the WHO classification. However, tissue analysis for NF2 is not routine in clinical practice. NF2 mutations are not believed to contribute to malignant progression [14]. NF2 is not an independent risk factor for recurrence, therefore as a prognostic marker of malignancy and recurrence NF2 does not appear to be useful.

However, NF2 appears to be a useful focus for targeted therapies. When examining clinical implications of NF2, Brastianos et al have defined a NF2 specific treatment arm testing FAK inhibitors [17] in the ongoing Alliance A071401 clinical trial. FAK inhibitors for NF2 mutations demonstrate excellent tolerability and improved progression free survival (PFS) compared to controls that warrant further investigation in larger trials [17]. Brigatinib is a multiple tyrosine kinase inhibitor, which also affects FAK as one of its targets. Early research has demonstrated that it may be an effective treatment of NF2 deficient meningioma, however further investigation is required [16]. Merlin has a role in inhibition of mTOR tumour growth pathways. Inhibitors of mTOR such as vistusertib are currently under investigation and early results in aggressive subsets of meningioma show promise [18]. FAK and mTOR inhibitors are a promising advancement in targeted meningioma therapy, in individuals with NF2 associated or NF2 related meningioma.

## Telomerase Reverse Transcriptase

Already incorporated into WHO classification of glioma, Telomerase Reverse Transcriptase (TERT) promoter mutations extend telomeres to produce immortal cancer cells [3, 7]. TERT mutations are found in a minority of tumours overall. Mutations in TERT are most common in high grade meningioma [19], which contain fewer targetable mutations when compared to low grade meningioma, however, such mutations are predicted to be neoantigens [20]. TERT mutations have been correlated to a high neoantigen load in all cancer types [21]. Similarly, TERT promotor mutations are associated with an increased risk of malignant histopathological progression [22].

There is a growing body of research investigating the molecular biology of these tumours, including genomic, transcriptomic, metabolomic, proteomic, and methylomic profiling. Such advancements in molecular profiling of meningioma are providing greater accuracy in prognostication of tumours.

The presence of TERT promotor mutations is linked to poor prognosis, reduced time to progression and increased risk of malignant histopathological progression in meningioma [7, 22]. Identification of TERT promoter mutations would identify those patients at higher risk of recurrence following treatment and might prompt the use of more frequent MRI surveillance and clinical follow-up. The importance of TERT mutations as a prognostic factor is exemplified by its inclusion as a signifier of grade 3 tumours in the updated WHO classification system [3].

Clinically, TERT mutations raise a number of questions regarding treatment and alternative therapies. TERT mutations are associated with high risk of recurrence following radiotherapy, which brings into question the clinical utility of adjuvant radiotherapy in this patient cohort [25]. The development of an alternative targeted treatment would offer clinicians a solution to this dilemma but this is not yet available. High neoantigen load in high grade meningioma presents the opportunity for immunologic therapy targeting TERT associated neoantigens. Similarly, TERT promotor mutation associated with histopathological progression allows for prospective targeting of low-grade meningioma with this mutation using aggressive TERT immunologic therapy.

## Other Molecular Mutations

Recent studies have identified phosphoinositide 3-kinase (PI3K), hereditary haemochromatosis (HH) and tumour necrosis factor receptor associated factor (TRAF7) mutations as significant markers of recurrence risk [23]. Kruppel like factor 4 (KLF4) mutations are protective against recurrence [23]. PI3K demonstrates the earliest recurrence rate, and along with HH is correlated to multiple driver genes [23]. Definitive identification of optimal driver genes in these mutations would allow for prognostic stratification and classification of affected meningioma, e.g., PI3KH1047R and SMOL412F respectively [23]. Similarly, it would allow for further development of targeted treatment clinical trials [17].

Alternative molecular markers to identify clinically aggressive meningioma are still relatively unexplored. Several studies have been performed but there can be a discrepancy in the presence of mutations between studies due to varying cohort sizes [37-39]. DNA Topoisomerase II Alpha (TOP2A) labelling is associated with a shorter overall and progression free survival, whilst N-MYC downstream- regulated gene 2 (NDRG2) is established as a marker of tumour aggression [24,25]. Polycomb Repressive Complex 2 (PCR2) activity is increased in more aggressive meningioma [24]. Larger scale studies are needed to validate these biomarkers before they can be considered clinically useful and incorporated into the WHO classification.

## Transcriptomics

Patel et al performed primary transcriptome analysis of meningioma samples [26]. They found meningioma samples clustered into three clinically significant groups: Type A, B and C [26]. These clusters demonstrated significant differences in mitotic activity (MIB1)- highest in Type C [26]. Transcriptomal changes in the form of DREAM complex loss correlate with the higher MIB1 in Type C meningioma [26]. DREAM complex bound with RB-like proteins allows a cell to remain quiescent [26]. However, when associated with MYBL2 and FOXM1 the DREAM complex becomes activated and subsequently drives cell proliferation [26]. Elevated FOXM1 and MYBL2 is associated with more aggressive meningioma [26–28]. Identifying loss of repressive DREAM complex as a characteristic feature of high-grade meningioma, would allow clinicians to identify individuals most at risk of recurrence and tumour aggression. This information would guide follow up and treatment decisions.

Meningioma sample clustering was not associated with WHO classification as per the 2016 classification. Transcriptomic clustering samples displayed a longer PFS despite being classified as WHO grade 2 meningioma. Recurrent tumour samples were found to be of the same transcriptomic clustering of the original tumour. Identifying similarities between original tumour and recurrent tumour offers scientists an insight into the pathophysiology of meningioma recurrence. Similarly, identifying a common transcriptomic change across meningioma allows for treatments to be developed that could prevent or rapidly treat tumour recurrence (e.g. restricting MYBL2 or FOXM1 expression).

## Metabolomics

Metabolomics refers to the study of the metabolome – the biochemical profile of a cell or organism. Metabolomic research has been used in a range of different cancer types to identify diagnostic biomarkers, driver mutations and monitor disease progression.

A metabolomic study by Masalha W et al identified two clusters of meningioma samples marked by metabolite alterations that separated samples by WHO grades, proliferation and PFS [29]. Another study demonstrated that meningioma metabolome provides a way of identifying aggressive meningioma allowing for personalised treatment [30]. Identification of metabolites within tumour samples that could identify more aggressive meningioma and those more at risk of progression could allow clinicians to approach such tumours with a more aggressive surgical approach and follow patients more closely than those without.

Away from meningioma research, in pheochromocytoma, paraganglioma and breast cancer metabolomics have been used to detect driver mutations with indicative metabolite profiles [31]. The ability to detect optimal driver mutations within meningioma would allow for the development of targeted therapies and aid prognostic stratification [23].

## Methylome Profiling

The process of methylation has a number of important functions in both physiology and pathophysiology. It helps prevent expression of harmful intergenetic regions of DNA, plays an important role in regulating gene expression through variable methylation of CpG sites, and functional knockout studies in methylation regulating proteins have demonstrated its importance in normal CNS development [32]. Abnormal activity of methylation regulating proteins, such as DNA methyltransferases (DMNT), are implicated in meningioma pathogenesis [32]. Aberrant methylation results in gene silencing by blocking the transcription of genetic material [33]. Pro-oncogenic changes in DNA methylation occur in the initial stages of tumour formation, meaning it is an early indication of the disease process [33].

Early research into the role of methylation as a prognostic classifier for meningioma did not provide a significant improvement over the WHO classification [34]. However, it did demonstrate the feasibility of using methylation to classify tumours, thereby laying the groundwork for future studies. In 2017, Sahm et al published a methylation based classification and grading system of meningioma, based on multi-institutional data [35]. There are a number of key findings to highlight from this study. Firstly, using genome wide methylation signatures, meningioma were successfully distinguished from other primary brain tumours [35]. Next, application of hierarchal clustering broadly identified two cohorts of meningioma based on their methylation expression. Within these cohorts, a further six subgroups were identified and designated Methylation Classes (MC) ben-1, MC ben-2, MC ben-3, MC int-A, MC int-B, and MC mal [35]. Kaplan Meier analysis demonstrated a reduction in PFS from the benign (MC ben-1, ben-2, ben-3), to intermediate (MC int-A, int-B), and the malignant (MC mal) groups. Crucially, both the crude molecular classification and a combined version were shown to outperform the WHO classification system in predicting PFS [35]. This improvement reflects the ability of methylation to better distinguish genetically unstable ‘low grade’ and stable ‘high grade’ meningioma [2].

Currently, methylation analysis is not a widespread component of pathological meningioma tissue analysis, owing to limited access to facilities and the associated cost. However, as the technology improves, becoming cheaper and more widely available, analysis of meningioma methylation will provide a greater degree of accuracy when clinically stratifying risk of recurrence. Subsequently, patients may be better selected for adjuvant therapies and intensities of follow-up, leading to an improvement in disease management and patient experience. Methylomics have also been used to identify potential new treatments for meningioma. A gene enrichment study using methylation profiling demonstrated that patterns associated with tumour recurrence may be sensitive to Docetaxel, a chemotherapy agent already used in the treatment of other cancers [36]. Methylome profiling may prove useful in identifying systemic therapies for aggressive subsets of meningioma, beyond simply targeting specific driver mutations. Finally, multifaceted integrative molecular classification systems are superior to uni-dimensional pathological analysis, and methylome profiling forms a key component of these updated approaches [37].

## Feasibility

Despite the promising discoveries in meningioma classification there are still a number of challenges to integrating molecular diagnostics into the current WHO classification. The majority of which studies looked at molecular diagnostics have been performed on tumour samples from a retrospective cohort. This data needs prospective validation in order to confirm retrospective results. Similarly without effective pharmacotherapy (e.g. TERT targeted therapies), clinicians must balance the benefits of identifying relevant mutations with the risk of delayed patient time to diagnosis. Finally, in patients who are already diagnosed, it is necessary to rerun tests and gain more information on their tumour without being clear on the benefit this would have for the patient.

Away from the biological issues there are a number of practical issues that must be considered when assessing the feasibility of an integrated molecular classification. Hospital infrastructures may not have the capacity or technology to perform complex additional tests on patient samples, meaning testing must be outsourced. Outsourcing to commercial companies not only incurs a large cost but prolongs the time for results to be returned to treating clinicians. Simultaneously, outsourcing to companies with varying capacities could result in some results returning prior to others as demonstrated in other brain tumours (e.g. awaiting MGMT methylation status in glioma samples). Subsequently, patients have a delayed time to final diagnosis, longer waiting time and potentially raised anxiety. Clinicians have to deal with having a fragmented pathological report, and make difficult decisions around formal diagnosis and when to invite patients to clinic. To develop an integrated molecular classification system, multiple analysis techniques are required, including whole-exome sequencing, copy number, DNA methylation, and mRNA sequencing [37]. Researchers have attempted to correlate complex integrated molecular classification groups to more clinically practical methods, such as protein expression on immunohistochemistry [37]. However, further evidence is needed to justify the validity of these findings.

## Conclusions

Neuro-Oncology is currently in the process of a molecular renaissance. Translational research is providing new insights into how clinicians can more accurately group phenotypically alike meningioma. This review has highlighted some of the key molecular factors of interest. Genomic, transcriptomic, metabolomic, and methylomic analysis is able to provide more representative prognostication of tumours, compared to the conventional WHO classification. Although the newly updated WHO classification reflects the importance of appreciating molecular factors, it still produces overly homogenised groups of behaviourally dissimilar tumours. Integrated molecular classifications provide even greater degrees of prognostic ability, at the expense of further reduced clinical applicability. Uncovering meningioma molecular biology is also providing powerful insights into potential targeted therapies, which may further improve patient care should they prove successful.
